# Using Artificial Intelligence to Semi-Quantitate Coronary Calcium as an ‘Incidentaloma’ on Non-Gated, Non-Contrast CT Scans, A Single-Center Descriptive Study in West Michigan.

**DOI:** 10.51894/001c.89132

**Published:** 2023-12-05

**Authors:** Connor C. Kerndt, Rajus Chopra, Paul Weber, Amy Rechenberg, Daniel Summers, Thomas Boyden, David Langholz

**Affiliations:** 1 Internal Medicine Spectrum Health/Michigan State University College of Human Medicine; 2 Department of Cardiology Spectrum Health/Michigan State University College of Human Medicine; 3 Internal Medicine Spectrum Health/Michigan State University: College of Human Medicine; 4 Department of Cardiology Spectrum Health/Michigan State University:; 5 Department of Internal Medicine Spectrum Health/Michigan State College of Human Medicine

**Keywords:** Artificial intelligence, Coronary artery calcium, Coronary artery disease, CAD, CAC, CT-scan

## Abstract

**INTRODUCTION:**

Non-gated, non-contrast computed tomography (CT) scans are commonly ordered for a variety of non-cardiac indications, but do not routinely comment on the presence of coronary artery calcium (CAC)/atherosclerotic cardiovascular disease (ASCVD) which is known to correlate with increased cardiovascular risk. Artificial intelligence (AI) algorithms can help detect and quantify CAC/ASCVD which can lead to early treatment and improved outcomes.

**METHODS:**

Using an FDA-approved algorithm (NANOX AI) to measure coronary artery calcium (CAC) on non-gated, non-contrast CT chest, 536 serial scans were evaluated in this single-center retrospective study. Scans were categorized by Agatston scores as normal-mild (<100), moderate (100-399), or severe (≥400). AI results were validated by cardiologist’s overread. Patient charts were retrospectively analyzed for clinical characteristics.

**RESULTS:**

Of the 527 patients included in this analysis, a total of 258 (48.96%) had moderate-severe disease; of these, 164 patients (63.57%, p< 0.001) had no previous diagnosis of CAD. Of those with moderate-severe disease 135 of 258 (52.33% p=0.006) were not on aspirin and 96 (37.21% p=0.093) were not on statin therapy. Cardiologist interpretation demonstrated 88.76% agreement with AI classification.

**DISCUSSION/CONCLUSION:**

Machine learning utilized in CT scans obtained for non-cardiac indications can detect and semi-quantitate CAC accurately. Artificial intelligence algorithms can accurately be applied to non-gated, non-contrast CT scans to identify CAC/ASCVD allowing for early medical intervention and improved clinical outcomes.

## INTRODUCTION

Atherosclerotic cardiovascular disease (ASCVD) continues to be the leading causes of morbidity and mortality in the United States and affects up to 50% of the population greater than 50 years of age.^1^ A highly specific feature of coronary atherosclerosis is the presence of coronary artery calcium (CAC), which represents a chronic inflammatory and pathologic process of atherosclerotic plaque.^2^ The presence and quantification of CAC has been shown to be an important independent risk factor for predicting the risk of major adverse cardiovascular events in asymptomatic individuals and is traditionally done by performing ECG-gated, non-contrast chest CTs.^3^ Clinically, CAC not only helps physicians develop preventive strategies with the use of enhanced risk scores to more accurately predict the risk of cardiac events, but also improves patient adherence to therapies.^4^ Given the utility CAC scores provide, currently American College of Cardiology/American Heart Association guidelines from 2019 provide a class IIa recommendation to obtain CAC scores in select patient populations.^5^

In 2016, Society of Cardiovascular Computed Tomography/Society of Thoracic Radiology created guidelines recommending evaluation and reporting of CAC on all non-contrast chest CT examinations as a class I indication. In addition to reporting CAC, these guidelines also recommend that CAC should be estimated as either none, mild, moderate, or severe either based on ordinal scoring or visual estimation. Although numerous studies have proven acceptable CAC score correlation between gated and non-gated, non-contrast CT (NCCT) scans, the latter poses more challenges to CAC quantitation due to potential motion artifact.^6^ Furthermore, current methods of estimating CAC on NCCT scans pose potential for inter-observer variability.

The use of artificial intelligence (AI) provides a unique opportunity to automatically detect and quantify significant CAC on NCCT’s to identify high risk individuals using deep learning end-to-end algorithms.^7^ AI can help improve speed in CAC score calculation, allow for prompt diagnosis and ultimately improve downstream patient care.^8^ Although several applications of AI have received FDA approval for clinical use, the American College of Cardiology and Society of Cardiovascular Computed Tomography do not have a formal consensus regarding ethical and appropriate application of AI in clinical practice.

In this study, expectations for AI technology were validated by a small panel of our institution’s cardiologists with accuracy and reproducibility to identify those with moderate or severe CAC and ultimately help implement preventive therapies to prevent future coronary related events. Early identification can allow for risk factor modification by implementing adequate glycemic control, blood pressure management, lipid reduction, and platelet inhibition. In this study, AI algorithms were utilized to accurately quantify and detect CAC. The goal of the project was to utilize AI on non-cardiac indicated CT-scans to detect asymptomatic patients with high degrees of CAC, to then expedite the consideration and likely initiation of risk reducing therapies.

## METHODS

Patient Selection and Materials: This study was a single-center retrospective study enrolling patients undergoing non-gated, non-contrast chest CT scans indicated for various reasons other than presurgical coronary artery bypass graft evaluation to be interpreted by the AI technology. As a numerical study, AI was utilized to only semi-quantitate CAC without assessment on clinical impact. An Institutional Review Board (IRB) approval was obtained prior to enrollment. Patients were enrolled from August 2020 through September 2020, until 536 consecutive CT-scans occurred. Patients were screened based upon established inclusion and exclusion criteria. The specific inclusion criteria screened patients for axial CT scans which included the entire cardiac silhouette of any gender and at least 30 years of age. CT scans were obtained by Philips, GE, and Siemens scanners and required a slice thickness of 0.1-3.1mm, slice interval of 0.1-3.1mm, and slice interval had to be equal to or less than the slice thickness. The exclusion criteria excluded patients with a scan consisting of less than 20 slices, scans that did not fully encircle the cardiac silhouette, CT attenuation correction, negative window center, contrast administration, gated CT scans, and CT scans that were ordered to assess for coronary artery disease (CAD). Repeat studies of the same patient were excluded so as only to include the first study in individuals duplicate scans, to not skew the data. In total, 6 scans were omitted due to uninterpretable imaging artifacts and 3 studies did not include enough of the cardiac silhouette to be assessed for CAC. Thus 527 scans were included in the analysis. Since study results were not going to be used in clinical care for the patients, the Institutional Review Board waived informed consent for this study.

### Coronary Artery Calcium Scoring

Coronary artery calcium scoring was performed using software from the Israeli machine-learning radiology firm *Nanox* to interpret and quantify CAC within a numerical score range. This non-ECG gated non-contrast coronary artery calcium scoring algorithm had already received FDA clearance and was designed to automatically calculate a patient’s Agatston equivalent coronary artery calcium score from NCCT scans to identify a patient’s coronary artery disease burden.^9^ CAC CT post-processing algorithms and software automated the estimation and reported CAC using the Agatston method.^10^ Once interpreted by the AI software, a single, independent cardiologist reviewed all CT scans to evaluate for accuracy and agreement of CAC severity. The AI CAC scores were not blinded from the cardiologist prior to evaluation. The reading cardiologist evaluated the degree of CAC using Agatston scores that were semi-quantitated based on prior experience. The cardiologist reviewed CAC highlighted by AI which was outside of the normal coronary artery distribution or scans which did not include the entire cardiac silhouette to accurately assess the degree of CAC. Each scan was then provided an independent impression from the validating cardiologist demonstrating agreement, disagreement, or cannot determine.

### Data Collection and Statistical Analysis

Following CAC Agatston score categorization by the AI algorithm, demographic and clinical information were collected for all patients. Specifically, the presence or absence of a prior diagnosis of CAD and the presence of significant cardiovascular risk factors (such as diabetes mellitus, hyperlipidemia, systemic hypertension, smoking status, previous heart catheterization, and family history of myocardial infarction) were documented. Demographic data including age, sex, and ethnicity were recorded for each of the patients. At the time CT scans were performed, it was recorded whether the patient was currently prescribed aspirin, statin therapy, or antihypertensive medications. Statistical analysis of the data was performed using Chi-squared and Fisher’s exact test to determine statistical significance between the moderate and severe groups based upon CAC Agatston scores.

## RESULTS

*Demographics:* In total, 527 scans attained inclusion based on the criteria outlined above. Of 527 scans, 269(51.04%) had normal-mild Agatston scores (0-100), 109(20.68%) demonstrated moderate coronary calcium scores (101-399), and 149(28.27%) demonstrated severe coronary calcium (≥400). These 527 scans detected 258 patients (48.96%) with moderate-severe degrees of coronary calcium, which further analysis occurred upon. This cohort consisted of 133(51.55%) males and 125(48.45%) females (table 1a).

**Table 1a.** Cohort Description of Patient with Moderate-Severe AI Detected CAC and Cardiologist AI Interpretation Agreement

**Variable**	**Label**	**Moderate (109)**	**Severe (149)**	**Overall (258)**	**p-value**	**Test**
Gender	F	59(54.13)	66(44.3)	125(48.45)	0.119	Chisq
	M	50(45.87)	83(55.7)	133(51.55)		
Ethnicity	Caucasian	102(93.58)	138(92.62)	240(93.02)	0.818	Fisher
	African/American	3(2.75)	5(3.36)	8(3.1)		
	Asian	1(0.92)	4(2.68)	5(1.94)		
	Hispanic	1(0.92)	1(0.67)	2(0.78)		
	Other	2(1.83)	1(0.67)	3(1.16)		
Caucasian	No	7(6.42)	11(7.38)	18(6.98)	0.765	Chisq
	Yes	102(93.58)	138(92.62)	240(93.02)		
Smoking	No	68(62.39)	100(67.11)	168(65.12)	0.431	Chisq
	Yes	41(37.61)	49(32.89)	90(34.88)		
Cath	No	92(84.4)	81(54.36)	173(67.05)	<0.001	Chisq
	Yes	17(15.6)	68(45.64)	85(32.95)		
Diabetes Mellitus	No	82(75.23)	101(67.79)	183(70.93)	0.193	Chisq
	Yes	27(24.77)	48(32.21)	75(29.07)		
Hypertension	No	41(37.61)	25(16.78)	66(25.58)	<0.001	Chisq
	Yes	68(62.39)	124(83.22)	192(74.42)		
HLD	No	24(22.02)	28(18.79)	52(20.16)	0.523	Chisq
	Yes	85(77.98)	121(81.21)	206(79.84)		
FH of MI	No	78(71.56)	105(70.47)	183(70.93)	0.849	Chisq
	Yes	31(28.44)	44(29.53)	75(29.07)		
On BP Meds	No	48(44.04)	33(22.15)	81(31.4)	<0.001	Chisq
	Yes	61(55.96)	116(77.85)	177(68.6)		
On Aspirin	No	68(62.39)	67(44.97)	135(52.33)	0.006	Chisq
	Yes	41(37.61)	82(55.03)	123(47.67)		
On Statin	No	47(43.12)	49(32.89)	96(37.21)	0.093	Chisq
	Yes	62(56.88)	100(67.11)	162(62.79)		
Current CAD	No	85(77.98)	79(53.02)	164(63.57)	<0.001	Chisq
	Yes	24(22.02)	70(46.98)	94(36.43)		
Hx of CAD prior to CT	No	85(77.98)	79(53.02)	164(63.57)	<0.001	Chisq
	Yes	24(22.02)	70(46.98)	94(36.43)		
Feedback	Disagree	18(16.51)	11(7.38)	29(11.24)	0.022	Chisq
	Agree	91(83.49)	138(92.62)	229(88.76)		

*LHC-Left Heart Catheterization, MI-Myocardial infarction, AI-Artificial intelligence

**Table 1b.** Age, Weight, and Cholesterol Panel of Patient with AI Interpreted Moderate-Severe CAC

	**Moderate (109)**			**Severe (149)**				
**Variable**	**N**	**Mean ± SD**	**Min - Max**	**N**	**Mean ± SD**	**Min - Max**	**p-value**	**Test**
Age, yr	109	68.04 ± 9.013	38 - 92	149	71.26 ± 9.275	52 - 94	0.01	Wilc(EV)
BMI, kg/m^2	107	29.81 ± 7.504	16.83 - 59.07	148	28.84 ± 7.301	15.29 - 53.48	0.223	Wilc(EV)
HDL	93	51.52 ± 19.69	12 - 105	141	50.61 ± 16.32	21 - 103	0.928	Wilc(EV)
LDL	93	95.85 ± 36.23	35 - 218	141	81.63 ± 34.19	5 - 216	0.003	Wilc(EV)
Total cholesterol	93	173.4 ± 41.73	96 - 298	141	156 ± 45.16	81 - 334	0.001	Wilc(EV)

Chi-squared analysis showed no significant difference between males and females in the moderate and severe groups (p=0.11)(table 1a/1b). This cohort was predominantly Caucasian, consisting of 240 Caucasian patients(93.02%), 8 African American patients(3.1%), 5 patients of Asian ancestry(1.94%), 2 Hispanic(0.78), and 8 who identified as other, which demonstrated no significant difference of calcium between the groups calculated via Fisher-Exact test (p=0.818)(table 1a). The moderate CAC group had an average age of 68.04 ± 9.013 years compared to the severe group 71.26 ± 9.275 years (p=0.01).

### Patient risk factors

Of the 258 patients with moderate-severe disease, 75(29.07%) patients had a history of diabetes and 183(70.93%) had no history of diabetes; furthermore, there was no significant difference of percentage of individuals with diabetes between the subgroups of moderate and severe CAC groups (p=0.193). History of hypertension was found in 192 patients (74.42%) with subgroup chi-squared analysis showing that patients in the severe CAC group had hypertension more frequently than those with moderate CAC (83.22% vs 62.39% and p<0.01)(table 1a/1b). In the moderate-severe group, 79.84% had a history of hyperlipidemia with 20.16% having no previous history (table 1a). Prior to scanning, 164 of 258 patients (63.57%) with moderate-severe scores had no previous history of CAD (table 1a, Table 2).

**Table 2a.** Cohort Description of Patient with AI Detected CAC with No Previous CAD History

**Variable**	**Label**	**Moderate (85)**	**Severe** **(79)**	**Overall (164)**	**p-value**	**Test**
Gender	F	46(54.12)	40(50.63)	86(52.44)	0.655	Chisq
	M	39(45.88)	39(49.37)	78(47.56)		
Ethnicity	Caucasian	81(95.29)	74(93.67)	155(94.51)	0.549	Fisher
	African/American	1(1.18)	2(2.53)	3(1.83)		
	Asian	0(0)	2(2.53)	2(1.22)		
	Hispanic	1(1.18)	0(0)	1(0.61)		
	Other	2(2.35)	1(1.27)	3(1.83)		
Caucasian	No	4(4.71)	5(6.33)	9(5.49)	0.739	Fisher
	Yes	81(95.29)	74(93.67)	155(94.51)		
Smoking	No	53(62.35)	53(67.09)	106(64.63)	0.526	Chisq
	Yes	32(37.65)	26(32.91)	58(35.37)		
Cath	No	82(96.47)	64(81.01)	146(89.02)	0.002	Chisq
	Yes	3(3.53)	15(18.99)	18(10.98)		
Diabetes Mellitus	No	67(78.82)	60(75.95)	127(77.44)	0.66	Chisq
	Yes	18(21.18)	19(24.05)	37(22.56)		
Hypertension	No	32(37.65)	18(22.78)	50(30.49)	0.039	Chisq
	Yes	53(62.35)	61(77.22)	114(69.51)		
HLD	No	19(22.35)	26(32.91)	45(27.44)	0.13	Chisq
	Yes	66(77.65)	53(67.09)	119(72.56)		
FH of MI	No	64(75.29)	60(75.95)	124(75.61)	0.922	Chisq
	Yes	21(24.71)	19(24.05)	40(24.39)		
On BP Meds	No	39(45.88)	24(30.38)	63(38.41)	0.041	Chisq
	Yes	46(54.12)	55(69.62)	101(61.59)		
On Aspirin	No	62(72.94)	47(59.49)	109(66.46)	0.068	Chisq
	Yes	23(27.06)	32(40.51)	55(33.54)		
On Statin	No	40(47.06)	36(45.57)	76(46.34)	0.848	Chisq
	Yes	45(52.94)	43(54.43)	88(53.66)		
Current CAD	No	85(100)	79(100)	164(100)		< 2x2 table
Hx of CAD prior to CT	No	85(100)	79(100)	164(100)		< 2x2 table
Feedback	Disagree	15(17.65)	7(8.86)	22(13.41)	0.099	Chisq
	Agree	70(82.35)	72(91.14)	142(86.59)		

**Table 2b.** Age and Cholesterol Profile of Those with No Previous History of CAD with AI Detected CAC

	**Moderate (85)**			**Severe (79)**				
**Variable**	**N**	**Mean ± SD**	**Min - Max**	**N**	**Mean ± SD**	**Min - Max**	**p-value**	**Test**
Age, yr	85	67.75 ± 9.016	38 - 92	79	72.3 ± 9.513	52 - 94	0.002	t (EV)
BMI, kg/m^2	83	29.94 ± 8.043	16.83 - 59.07	78	28.03 ± 7.228	15.29 - 50.65	0.122	Wilc(EV)
HDL	72	51.67 ± 19.31	12 - 105	73	53.68 ± 16.38	26 - 97	0.306	Wilc(EV)
LDL	72	93.93 ± 34.28	35 - 187	73	88.62 ± 36.23	35 - 216	0.29	Wilc(EV)
Total cholesterol	72	171.6 ± 40.14	96 - 269	73	165.6 ± 48.38	81 - 334	0.166	Wilc(EV)

Of those with previous CAD history, CAC was typically more severe, 46.98% versus 22.02% (p<0.001) (table 1a/1b). Coronary angiograms were only previously performed in 85 patients (32.95%), coronary angiograms were more common in those with CTs of severe disease (45.64%) versus moderate disease (15.6%) (p<.001). Of the 258 patients with moderate-severe disease, only 123(47.67%) were on aspirin therapy. leaving 135(52.33%) without therapy; Subgroup analysis showed higher percentage of the severe group to be on aspirin with 55.03% versus 37.61% in the moderate group (p=0.01). Similarly, only 162 of 258(62.79%) were on statin therapy prior to CT scan and the remaining 37.21% not on therapy.

A sub-cohort of 164 patients had no previous history of ASCVD, but AI interpretation revealed moderate-severe CAC as described in table 2. Within this subset, 109(66.46%) patients were not on aspirin, including 47(59.49%) patients with severe CAC and 62(72.94%) with moderate CAC. Furthermore, 76(46.3%) patients were not on statin, including 36(45.57%) with severe CAC and 40(47.06%) with moderate CAC (Table 2).

### AI Agreement

AI CAC reading demonstrated agreement with cardiologist readings in 229 of 258 scans (88.76%), disagreement in 29 scans(11.24%). Subgroup analysis showed lower levels of agreement in the moderate group, 91 of 109 scans (83.49%), compared to the severe group, which had agreement in 138 of 149 scans (92.62%) (p=0.022). In the subcohort of patients without a prior history of CAD, total AI agreement was 86.59%, with 91.14% agreement in the severe group and 82.35% in the moderate group (Table 2).

## DISCUSSION

A multitude of different specialties have proven AI capable of significant clinical applicability including pathology, radiology, ophthalmology, and dermatology. Use of AI in these medical specialties has demonstrated practicality and utility as a screening tool to assist the physician’s efficiency and accuracy.^11^ This single-center, retrospective study was used to investigate the efficacy and utility of AI in the realm of cardiology via categorizing the degree of coronary calcium. CAC scoring can assist as a surrogate marker for CAD.^12^ CAC scoring has demonstrated reliability in assessing risk of major adverse cardiovascular events in clinical or subclinical CAD.^12^ While a CAC score of 0, does not necessarily rule out obstructive CAD, it would suggest that these patients are exceptionally rare(<5%).^12^ Herein, the authors describe AI’s ability to accurately identify and categorize degree of CAC, describe the patient population in which subclinical CAD was detected, and identify patients on suboptimal management.

The AI algorithm utilized in this single-center study demonstrated an overall agreement (88.76%) in the detection of CAC when comparing computer generated results and a single, trained cardiologist’s interpretation of the same CT scans. This agreement was even higher when evaluating severe disease (92.62%, p<.022). Previous studies utilizing AI to detect CAC have generated similar agreement in risk stratification (89.5%, p<0.001) when the analysis was verified by a trained cardiologist.^13^ Patients with large amounts of aortic calcification, specifically within the aortic valve annulus or ascending aorta were more prone to overestimation of CAC, which confounded AI interpretation.

In this study, 258(48.96%) patients had either moderate or severe CAC, of which 164(63.57%) did not have any known history of CAD. Previously unknown moderate-severe CAC was an incidental finding in 164(31.12%) of the 527 patients by applying AI as a screening tool; thus demonstrating its applicability, with little to no disadvantage.

The value of this tool is directly proportional to the ability to identify patients with subclinical CAD and thus lead to earlier risk-factor modification and treatment to slow disease progression. This study found over half (52.33%) of these patients with moderate-severe disease were not on aspirin and over a third (37.21%) were not on statin therapy; both of which would be indicated given the presence of ASCVD as estimated by CAC.^14^ This highlights a large patient population that may benefit from early detection of subclinical ASCVD, medical intervention and optimization, and reduced risk of major adverse cardiac events. Table 2a/b describes the population for which AI found moderate-severe CAC without previous history; this could describe the population for which patients may benefit most from CAC evaluation. Interestingly, a majority of patients without previous diagnosis in which CAC were detected had no history of diabetes (77.4% vs 22.56%)(Table 2a/b). While it’s difficult to extrapolate on this singular point, this finding suggests an opportunity for future research to understand the application of AI as a screening tool to detect CAD in non-diabetic patients.

While gated, non-contrast CT-scans are the preferred method for calculating CAC scores, non-gated, non-contrast CT-scans are performed with increasing volume for a myriad of non-cardiac indications, which allows an opportunity to detect subclinical coronary artery calcium/atherosclerotic cardiovascular disease. One study suggests that in the United States, >85 million CT scans were performed in 2012 and this number is expected to increase at approximately 6% per year; a significant portion likely to be containing the heart.^15^ This provides ample opportunity for early detection of CAC/ASCVD. Use of AI could standardize the detection of CAC and potentially curtail some of the labor that evaluation for CAD on NCCT scans would require, thus potentially improving efficiency. Furthermore, scans could be retroactively reevaluated with AI software to efficiently assess for CAC. Utilizing AI as an efficient tool to detect and quantify CAC could aid risk factor assessment and stratification during shared decision-making between providers and patients.^16^

The benefits of early detection of ASCVD at a health system level would have substantial population health impact on disease progression and downstream clinical outcomes.^17^ To increase awareness of the AI generated CAC findings, and promote initiation of indicated medical therapy, this study’s institution is piloting an automated alert in the electronic medical record with these findings. This would incorporate AI generated impressions as a diagnosis recommendation to ensure that these incidental findings are considered in outpatient management.

### Study Limitations

As a single-center study conducted on a primarily Caucasian population, there may be limited generalizability and widespread application of this data. This is a numerical research study and thus will only semi-quantitate CAC to be used as an adjunct in clinical decision-making. Since the CT scans interpreted were non-ECG gated studies, the likelihood of missing calcium (having a false negative) is higher than an ECG-gated study. The non-gated nature of these scans may predispose a small number of studies to cardiac motion artifact, thus making some studies uninterpretable. Future studies are needed to evaluate further applicability and validate new and improved AI algorithms to standardize the detection of CAC.

Although the *Nanox* AI algorithm is most efficient at interpreting CAC in the true negative population, it has demonstrated utility in the general population of patients requiring non-gated CT imaging of the chest.^18^ Since the validating cardiologist was not blinded from the AI CAC results this study could have been influenced by conformation bias. Another limitation of this study is that there was only one cardiologist involved in the independent analysis of agreement which may bias interpretation. Additionally, this study did not incorporate interpretation from a radiology perspective.

It is important to note that this study was designed to interpret CT scans of patients who required CT imaging of the chest. This study did not include a randomized control population of healthy patients. While this can be seen as a limitation of the study, it may also suggest that these patients undergoing the CT scan have more comorbidities, which place them at a higher risk for CAD (table 1a/1b). Arguably, this would make the “incidental” finding of CAC even more clinically relevant as compared to a healthy population, especially given that ischemic heart disease continues to be a leading cause of death.^19^

## CONCLUSIONS

At no significant additional radiation exposure to the patient, AI can be used to reliably screen for CAC/ASCVD with 88.76% agreement. The utility of AI can detect undiagnosed CAC/ASCVD to initiate therapy and reduce the risk of cardiac events. Further studies are needed to determine the suspected beneficial impacts such as cost savings, mortality and quality of life.

### Conflict of Interest

None
